# miR-132-y Targets *YAP1* and Modulates Sertoli Cell Viability-Associated Transcriptional Responses in Southdown × Hu F_1_ Sheep

**DOI:** 10.3390/biom16070995

**Published:** 2026-07-07

**Authors:** Binpeng Xi, Zengkui Lu, Rui Zhang, Lina Zhu, Miaoshu Zhang, Xuejiao An, Yaojing Yue

**Affiliations:** 1 Key Laboratory of Animal Genetics and Breeding on the Tibetan Plateau, Ministry of Agriculture and Rural Affairs, Lanzhou Institute of Husbandry and Pharmaceutical Sciences, Chinese Academy of Agricultural Sciences, Lanzhou 730050, China; w5562080w@163.com (B.X.); luzengkui@caas.cn (Z.L.); zhangrui@caas.cn (R.Z.); 19530465839@139.com (L.Z.); xbprl1008@163.com (M.Z.); 2 Sheep Breeding Engineering Technology Research Center, Chinese Academy of Agricultural Sciences, Lanzhou 730050, China

**Keywords:** *YAP1*, miR-132-y, Sertoli cells, Hippo signaling pathway, F_1_ hybrid of Southdown × Hu sheep

## Abstract

Sertoli cells are essential for testicular development and spermatogenesis, but the post-transcriptional mechanisms regulating their function in sheep remain incompletely understood. This study investigated the regulatory relationship between miR-132-y and Yes-associated protein 1 (*YAP1*), a core effector of the Hippo pathway, in primary Sertoli cells isolated from Southdown × Hu F1 sheep. Target prediction and dual-luciferase reporter assays supported a direct interaction between miR-132-y and the YAP1 3′ untranslated region. *YAP1* overexpression was associated with increased CCK-8-based cell viability and altered mRNA expression of selected viability-associated, *YAP1*-related, and Sertoli cell function-associated genes, whereas *YAP1* silencing showed opposite trends. Conversely, miR-132-y overexpression reduced *YAP1* mRNA abundance and was associated with decreased CCK-8-based cell viability and corresponding transcriptional changes, while miR-132-y inhibition produced the opposite pattern. Rescue experiments showed that ectopic *YAP1* expression partially attenuated miR-132-y-associated changes. Overall, these findings provide in vitro, cell-based evidence that miR-132-y targets *YAP1* at the transcript level and is associated with viability-related transcriptional responses in sheep Sertoli cells.

## 1. Introduction

Hu sheep are widely recognized in China for their high prolificacy, and their outstanding reproductive performance has made them an important resource in genetic improvement and breeding programs [1]. Southdown sheep, when used as terminal sires, contribute favorable production traits to their offspring, particularly improved post-weaning growth performance and meat quality [2]. Accordingly, the F1 offspring generated from Southdown × Hu crosses exhibit superior meat quality and reproductive performance relative to the parental breeds [3,4]. Because normal testicular development is fundamental to male fertility, Sertoli cells (SCs) are indispensable for the establishment and maintenance of the spermatogenic microenvironment [5,6,7,8]. Through structural, nutritional, and regulatory support to developing germ cells, SCs critically influence spermatogenesis and testicular function. Therefore, clarifying the molecular mechanisms that regulate SC biology is important for understanding male reproductive development in mammals.

Non-coding RNAs, especially microRNAs (miRNAs), are important post-transcriptional regulators in the testis and participate in the functional regulation of Sertoli cells, Leydig cells, and germ cells [9,10,11,12]. Declining semen quality and male infertility have also been associated with molecular alterations in small noncoding RNAs, which may not be fully captured by conventional semen parameters. Recent evidence indicates that sperm small noncoding RNAs and sperm nuclear basic proteins can reflect environmental impacts on male germ cells, while miRNA dysregulation has been linked to spermatogenesis-related disorders in infertile men [13,14]. In SCs, miRNAs have been implicated in the control of cell survival, proliferation, differentiation, and other biological processes through their interactions with target mRNAs. However, the specific miRNAs and downstream regulatory axes that govern SC function in livestock species remain insufficiently understood. Among these molecules, miR-132 has attracted increasing attention because of its involvement in cell proliferation and differentiation [15]. In a vinclozolin-induced reproductive injury model, *YAP1* was identified as a direct target of miR-132, and increased miR-132 expression was associated with reduced *YAP1* abundance and abnormal male reproductive phenotypes, including penile malformation and reduced testis size [16]. These observations suggest that the miR-132–*YAP1* axis may participate in male reproductive development. *YAP1* is a core effector of the Hippo signaling pathway and is widely recognized as an important regulator of cell growth, survival, and organ size [17]. Although *YAP1*-dependent regulatory mechanisms, including its interaction with *LATS2* in adipogenesis, have been described in other biological contexts [18], its role in ruminant Sertoli cells remains poorly defined.

In the present study, SCs isolated from Southdown × Hu F1 sheep were used as an in vitro model to investigate the relationship between miR-132-y and *YAP1* in sheep Sertoli cells. Based on in silico target prediction, *YAP1* was identified as a candidate target of miR-132-y, and the predicted interaction was further supported by dual-luciferase reporter assays. We further examined the effects of *YAP1* overexpression or silencing, as well as miR-132-y overexpression or inhibition, on SC viability and on the expression of genes related to *YAP1*-associated signaling and SC function. In addition, rescue experiments were performed to assess whether *YAP1* may mediate, at least in part, the effects of miR-132-y in this cell model. Collectively, this study provides evidence for a miR-132-y–*YAP1* regulatory relationship in sheep SCs and offers a basis for further investigation of post-transcriptional regulation in testicular somatic cells.

## 2. Materials and Methods

### 2.1. Experimental Animals and Samples

All animal procedures were reviewed and approved by the Animal Management and Ethics Committee of the Lanzhou Institute of Husbandry and Veterinary Medicine, Chinese Academy of Agricultural Sciences (Approval No. 0231447; approval date: 19 November 2023). All procedures involving animals were performed in accordance with the institutional guidelines for animal care and welfare. Testicular tissues were aseptically collected from clinically healthy 4-month-old male Southdown × Hu F1 sheep at Qing huan Mutton Sheep Breeding Company, Gansu Province, China. The age of each animal was verified using breeding records. Four clinically healthy 4-month-old male Southdown × Hu F1 sheep were used as donor animals in this study. Primary Sertoli cells (SCs) were independently isolated and cultured from the testicular tissue of each donor animal. Unless otherwise stated, biological replicates represented independent Sertoli cell preparations derived from different donor animals and processed independently for subsequent culture, transfection, RNA extraction, RT-qPCR, and cell viability assays. HEK-293T cells used for dual-luciferase reporter assays were obtained from our laboratory and maintained in high-glucose DMEM supplemented with 10% FBS and 1% penicillin–streptomycin (100×; Solarbio, Beijing, China) solution at 37 °C in a humidified incubator containing 5% CO_2_. Cells were passaged at approximately 80–90% confluence and used for transfection during the logarithmic growth phase.

### 2.2. Isolation, Purification, and Culture of Primary Sertoli Cells from Southdown × Hu F1 Sheep

Testes were collected following castration under general anesthesia. General anesthesia was induced by intramuscular injection of diazepam (410 mg; Jining Ankang Pharmaceutical Co., Ltd., Jining, China) and scopolamine (90.3 mg; ChemeGen, Shanghai, China), followed by intravenous administration of thiopental sodium (10–20 mg/kg; Shanghai SPH New ASIA Pharmaceutical Co., Ltd., Shanghai, China). After collection, the tunica albuginea was aseptically removed, and the testicular tissue was washed three times with phosphate-buffered saline (PBS) containing antibiotics.

The tissue was mechanically minced and subjected to two-step enzymatic digestion. Briefly, tissue fragments were first digested with collagenase IV at 37 °C for 50 min, followed by digestion with 0.25% trypsin for 15 min. The concentration of collagenase IV used was 1 mg/mL. Digestion was terminated by adding complete medium. The resulting cell suspension was sequentially filtered through 100-, 200-, and 300-mesh sieves, centrifuged at 1000× *g* for 5 min, and washed three times with PBS containing 1% antibiotics.

Primary cells were cultured in DMEM/F-12 complete medium supplemented with 10% fetal bovine serum (FBS) and 1% antibiotics at 37 °C in a humidified atmosphere containing 5% CO_2_. Sertoli cells were enriched by repeated hypotonic treatment and differential adhesion. When cells reached approximately 80% confluence, they were detached using 0.05% trypsin. This purification procedure was repeated 3–4 times to obtain an enriched population of immature SCs.

### 2.3. Identification of Primary SCs from F1 Hybrid of Southdown × Hu Sheep

Purified SCs were identified by immunofluorescence staining, Oil Red O staining, and alkaline phosphatase (ALP) staining, as described in the original experimental workflow. The isolated cells showed the typical bipolar morphology of SCs, and cell purity in the present study exceeded 85%, consistent with the subsequent characterization results.

For immunofluorescence staining, purified SCs were seeded in 6-well plates at a density of 2 × 10^5^ cells/well, allowed to adhere overnight, and fixed with 4% paraformaldehyde for 30 min at room temperature after PBS washing. Cells were permeabilized with 0.1% Triton X-100 (Thermo Scientific, Waltham, MA, USA, 28313) for 10 min, blocked with 5% bovine serum albumin (BSA) for 30 min, and incubated overnight at 4 °C with primary antibodies against GATA4 (Bioss, Beijing, China, bs-1778P) and SOX9 (Bioss, Pbs-4177R) at a dilution of 1:200. After washing with PBS, cells were incubated with a Cy3-conjugated secondary antibody (1:500) for 90 min at 37 °C in the dark. Nuclei were counterstained with DAPI for 5 min, and fluorescence images were acquired under a fluorescence microscope.

For Oil Red O staining, cells were fixed and processed using an Oil Red O staining kit (Beijing Solarbio Science & Technology Co., Ltd., Beijing, China., G1261) according to the manufacturer’s instructions, followed by hematoxylin counterstaining. For ALP staining, cells were fixed in 4% paraformaldehyde and stained using an ALP detection kit (Beijing Solarbio Science & Technology Co., Ltd., G1480) according to the manufacturer’s instructions. ALP-positive cells were identified by blue–purple membrane-associated staining under an inverted microscope.

### 2.4. Construction of YAP1 Gene Overexpression and Interference Vectors

Three siRNAs targeting the coding sequence (CDS) of ovine *YAP1* (si-*YAP1*-1, si-*YAP1*-2, and si-*YAP1*-3), together with a negative control siRNA (si-NC), were designed. The *YAP1* overexpression plasmid pcDNA3.1(+)-*YAP1* and the corresponding empty vector pcDNA3.1(+) were commercially synthesized by Genewiz (GENEWIZ, Suzhou, China). Plasmids were extracted using a low-endotoxin mini prep kit (DP103-02; TIANGEN Biotech Co., Ltd., Beijing, China). Detailed sequences are provided in Supplementary Table S1.

### 2.5. Construction of YAP1 3′-UTR Wild-Type and Mutant Recombinant Plasmids

Potential miRNA-binding sites in the ovine *YAP1* 3′UTR were predicted using TargetScan (version 7.2). Wild-type (WT) and mutant (MUT) *YAP1* 3′UTR fragments were generated by molecular cloning. In the mutant construct, the predicted miRNA-binding site was replaced with a reverse-complementary sequence to disrupt the interaction. The *YAP1* 3′UTR fragments were directionally cloned into the pmirGLO luciferase reporter vector using Xho *I* and Sal *I* restriction sites. All constructs were verified by sequencing and double enzyme digestion. The final recombinant plasmids were designated *YAP1* 3′UTR-WT and *YAP1* 3′UTR-MUT. Full sequence information is provided in Supplementary Figure S1.

### 2.6. Design and Synthesis of miRNA Mimics and Inhibitors

Based on the mature ovine miR-132-y sequence, the corresponding miR-132-y mimic and inhibitor were designed and synthesized. For the mimic duplex, the sense strand corresponded to the mature miR-132-y sequence, and the antisense strand was generated as the reverse complement of the sense strand excluding the final two nucleotides, followed by addition of a 3′ UU overhang. The inhibitor sequence was designed as the full reverse complement of mature ovine miR-132-y. Detailed sequences are listed in Supplementary Table S2. Corresponding negative controls were used in all transfection experiments.

### 2.7. Cell Transfection

SCs were seeded in 6-well plates at a density of 2 × 10^5^ cells/well in DMEM/F-12 medium supplemented with 10% FBS. When cells reached approximately 40% confluence, they were washed three times with PBS (3 min each). Transfections were performed using Lipofectamine 3000 (nvitrogen; Thermo Fisher Scientific, Carlsbad, CA, USA) according to the manufacturer’s instructions. For YAP1 overexpression, 2.5 μg pcDNA3.1(+)-*YAP1* plasmid or empty vector was diluted in 125 μL Opti-MEM, and 5 μL Lipofectamine 3000 was diluted separately in 125 μL Opti-MEM. The two solutions were combined, incubated for 15 min at room temperature, and added to cells. For *YAP1* interference, siRNA or si-NC was transfected at a final concentration of 20 nM using the same protocol. The total volume in each well was adjusted to 2 mL with Opti-MEM. After 6 h, the transfection medium was replaced with complete culture medium containing 1% antibiotics, and cells were harvested 48 h post-transfection.

For miRNA experiments, SCs were transfected with miR-132-y mimic, mimic negative control, miR-132-y inhibitor, or inhibitor negative control under the same conditions. The final concentrations of the mimic and inhibitor were 10 nM, respectively. For the dual-luciferase reporter assay, HEK-293T cells were seeded in 24-well plates at a density of 1 × 10^5^ cells/well and transfected when they reached approximately 70–80% confluence. For each well, 1 μg of pmirGLO reporter plasmid containing the wild-type or mutant *YAP1* 3′UTR was diluted in 50 μL Opti-MEM. In parallel, 2 μL Lipofectamine 3000 reagent was diluted in 50 μL Opti-MEM. The diluted DNA and Lipofectamine 3000 solutions were gently mixed and incubated for 15 min at room temperature before being added dropwise to each well. The final transfection volume in each well was adjusted to 500 μL. Cells were co-transfected with the reporter plasmid and miR-132-y mimic at a final concentration of 50 nM or miR-132-y inhibitor at a final concentration of 100 nM. The corresponding mimic negative control or inhibitor negative control was used under the same conditions. After 4 h, the transfection medium was replaced with complete culture medium, and cells were harvested 48 h after transfection for the dual-luciferase reporter assay. The effectiveness of *YAP1* overexpression, *YAP1* knockdown, and miR-132-y mimic/inhibitor transfection was evaluated by RT-qPCR analysis of the corresponding target transcripts 48 h after transfection. No fluorescence-labeled control or flow cytometry-based assay was used to directly quantify the absolute transfection efficiency.

Rescue experiments were performed using four transfection groups: (1) miR-132-y mimic + pcDNA3.1(+)-*YAP1*, (2) miR-132-y mimic + empty vector, (3) mimic negative control + pcDNA3.1(+)-*YAP1*, and (4) mimic negative control + empty vector. For all transfection experiments, the corresponding negative controls were transfected under the same conditions as the treatment groups. The siRNA negative control (si-NC) was a non-targeting siRNA sequence used as the control for *YAP1* knockdown. The mimic negative control (mimic NC) was a scrambled RNA duplex used as the control for the miR-132-y mimic, whereas the inhibitor negative control (inhibitor NC) was a scrambled antisense oligonucleotide used as the control for the miR-132-y inhibitor. The empty pcDNA3.1(+) vector was used as the plasmid control for *YAP1* overexpression. Unless otherwise stated, the term “control group” refers to the corresponding negative control group transfected under identical experimental conditions.

### 2.8. Dual-Luciferase Reporter Assay

HEK-293T cells were collected 48 h after transfection. Cells were washed twice with PBS and lysed with 100 μL precooled passive lysis buffer (1 × PLB). Plates were shaken at 200 rpm for 10 min at room temperature to ensure complete lysis. A 100 μL aliquot of lysate was transferred to a 96-well plate. Firefly luciferase activity was measured after addition of 50 μL Luciferase Assay Reagent II (LAR II), followed by measurement of Renilla luciferase activity after addition of an equal volume of Stop & Glo reagent (Promega, Corporation, Madison, WI, USA). Relative luciferase activity was calculated as the ratio of firefly to Renilla luminescence.

### 2.9. Cell Counting Kit-8

Cell viability was assessed using the Cell Counting Kit-8 (CCK-8, Dojindo Laboratories, Kumamoto, Japan). Logarithmically growing SCs were seeded into 96-well plates at a density of 1 × 10^4^ cells/well in 100 μL culture medium and cultured at 37 °C in 5% CO_2_ for 24 h to allow cell adhesion. Cells were then subjected to the corresponding transfection treatments. At 24, 48 and 72 h post-transfection, 10 μL CCK-8 reagent was added to each well, followed by incubation at 37 °C in the dark for 120 min. Absorbance was measured at 450 nm using a microplate reader (BioTek Instruments, Winooski, VT, USA). Each experiment included three independent biological replicates, and each condition within one biological replicate contained six technical replicate wells.

### 2.10. Total Cellular RNA Extraction and RT-qPCR

First-strand cDNA was synthesized using the Transcriptor First Strand cDNA Synthesis Kit (Roche Diagnostics GmbH, Mannheim, Germany) according to the manufacturer’s instructions. RT-qPCR was performed on a CFX96 Real-Time PCR System (Bio-Rad, Inc., Hercules, CA, USA) using a SYBR Green-based qPCR assay (Bio-Rad Laboratories, Inc., Hercules, CA, USA). Each 20 μL reaction contained 10 μL of 2× SYBR Green qPCR Master Mix, 0.4 μL of forward primer, 0.4 μL of reverse primer, 2 μL of diluted cDNA template, and 7.2 μL of nuclease-free water. The final concentration of each primer was 0.2 μM. The amplification program was as follows: initial denaturation at 95 °C for 30 s, followed by 40 cycles of 95 °C for 5 s and 60 °C for 30 s. A melting curve analysis was performed from 65 °C to 95 °C to confirm amplification specificity. No-template controls (NTCs) and no-reverse-transcription controls (no-RT controls) were included in each run to monitor reagent contamination and genomic DNA carryover. Primer sequences are listed in Table S1, and primer specificity was confirmed by melting curve analysis. U6 small RNA and β-actin were used as internal controls for miRNA and mRNA quantification, respectively. Relative expression levels were calculated using the 2^−ΔΔCt^ method, and each sample was analyzed in technical triplicate.

### 2.11. Statistical Analysis

Statistical analyses were performed using GraphPad Prism version 9.0. Data are presented as mean ± standard deviation (SD). Unless otherwise stated, each experiment included three independent biological replicates. Each biological replicate corresponded to an independently prepared primary Sertoli cell culture derived from a different donor animal and processed independently for culture, transfection, RNA extraction, RT-qPCR, dual-luciferase reporter assay, and CCK-8 assay. Technical replicates were performed within each biological replicate as indicated for each assay. For the CCK-8 assay, each biological replicate contained six technical replicate wells per condition. For RT-qPCR, each biological replicate was analyzed in technical triplicate. For comparisons between two groups, an unpaired Student’s *t*-test was used. For comparisons among more than two groups, one-way ANOVA followed by Tukey’s post hoc multiple-comparison test was applied. The specific statistical test used for each figure panel is indicated in the corresponding figure legend. A value of *p* < 0.05 was considered statistically significant.

## 3. Results

### 3.1. Isolation, Culture, and Characterization of SCs from F_1_ Hybrid of Southdown × Hu Sheep

**Figure 1:**
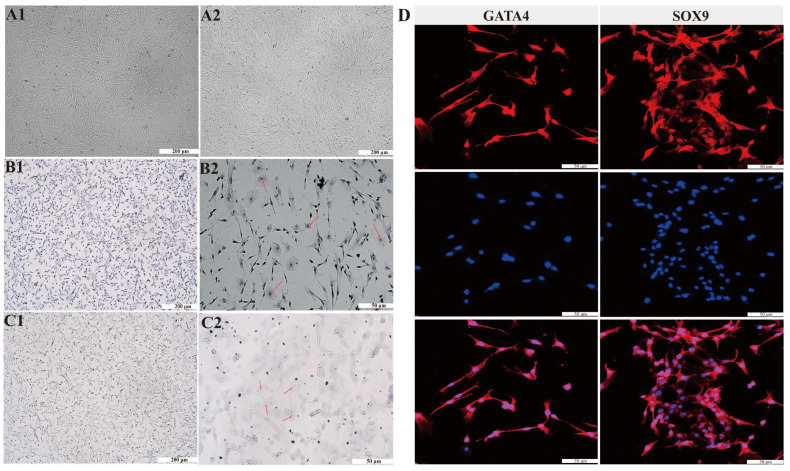
Isolation, morphological characterization, and identification of primary Sertoli cells from Southdown × Hu F1 sheep. (**A1**,**A2**) Morphology of primary Sertoli cells after purification and culture, showing a relatively uniform cell population with typical elongated or bipolar morphology. (**B1**,**B2**) Oil Red O staining of cultured cells. Intracellular lipid droplets were observed in the cytoplasm (red arrows). (**C1**,**C2**) Alkaline phosphatase (ALP) staining of cultured cells. Only weak positive signals were detected (red arrows), suggesting low contamination by peritubular myoid cells. (**D**) Immunofluorescence staining of Sertoli cell markers GATA4 and SOX9. Red fluorescence indicates GATA4 or SOX9 signal, and blue fluorescence indicates DAPI-stained nuclei. Scale bars: 200 μm in (**A1**,**B1**,**C1**); 50 μm in (**A2**,**B2**,**C2**,**D**).

Primary SCs were isolated by differential adhesion combined with hypotonic treatment and 0.05% trypsin digestion to reduce contamination from spermatogenic and interstitial cells. After 48 h of purification, the proportion of SCs exceeded 85%. By the third passage, the cells displayed a relatively uniform morphology and stable growth characteristics (Figure 1(A1,A2)). Cell identity and enrichment were further evaluated by morphological and staining analyses. The isolated cells exhibited the typical bipolar morphology of SCs, with elongated cytoplasmic processes. Oil Red O staining showed the presence of intracellular lipid droplets with relatively weak staining intensity (Figure 1(B1,B2)). ALP staining revealed only minimal positive signals, suggesting low contamination by peritubular myoid cells (Figure 1(C1,C2)). In addition, immunofluorescence staining showed strong GATA4 and SOX9 signals in the cultured cells (Figure 1D), consistent with the characteristics of Sertoli cells. Together, these findings indicate that the isolated cell population was highly enriched for SCs and was suitable for subsequent in vitro experiments.

### 3.2. Association of YAP1 Overexpression or Silencing with Sertoli Cell Viability and Viability-Related Transcript Expression

**Figure 2:**
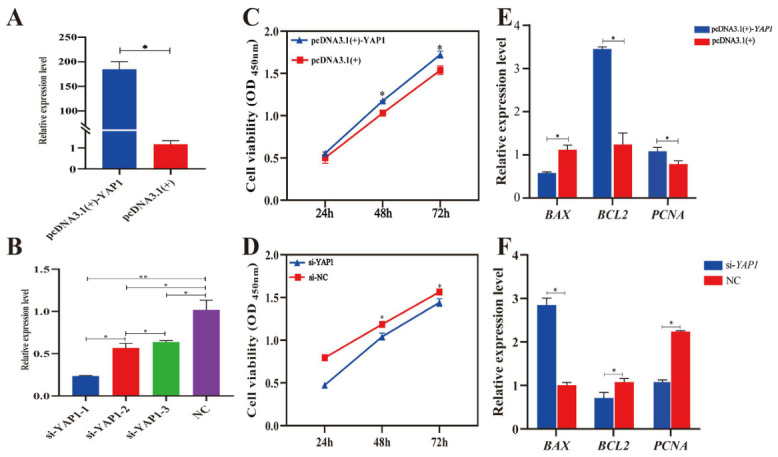
Effects of *YAP1* overexpression and silencing on Sertoli cell viability and mRNA expression of viability-associated genes, including *BAX*, *BCL2*, and *PCNA*, as assessed by RT-qPCR. (**A**) RT-qPCR analysis of *YAP1* mRNA expression after transfection with the *YAP1* overexpression plasmid pcDNA3.1(+)-*YAP1* or the empty vector pcDNA3.1(+). (**B**) RT-qPCR analysis of *YAP1* mRNA expression after transfection with three *YAP1* siRNAs (si-*YAP1*-1, si-*YAP1*-2, and si-*YAP1*-3) and the negative control (NC). (**C**) CCK-8 assay showing changes in cell viability after *YAP1* overexpression at 24, 48, and 72 h post-transfection. (**D**) CCK-8 assay showing changes in cell viability after *YAP1* silencing at 24, 48, and 72 h post-transfection. (**E**) RT-qPCR analysis of *BAX*, *BCL2*, and *PCNA* mRNA expression after *YAP1* overexpression. (**F**) RT-qPCR analysis of *BAX*, *BCL2*, and *PCNA* mRNA expression after *YAP1* silencing. Data are presented as mean ± SD from three independent biological replicates. In panels (**A**,**C**–**F**), statistical significance was assessed by Student’s *t*-test between the two indicated groups. In panel B, statistical significance was assessed by one-way ANOVA followed by a post hoc multiple-comparison test. Data are presented as mean ± SD from three independent biological replicates. Panels (**A**,**C**–**F**) were analyzed using an unpaired Student’s *t*-test between the two indicated groups at each time point or for each gene. Panel (**B**) was analyzed using one-way ANOVA followed by Tukey’s post hoc test. *p* < 0.05 was considered statistically significant. Data are presented as mean ± SD. * *p* < 0.05 indicate statistically significant differences; ** *p* < 0.01.

RT-qPCR performed 48 h after transfection confirmed the effectiveness of *YAP1* overexpression and silencing. Compared with the corresponding control, *YAP1* mRNA expression was significantly increased in the pcDNA3.1(+)-*YAP1* group (*p* < 0.05; Figure 2A). Among the three interference fragments, si-*YAP1*-1 showed the strongest inhibitory effect on *YAP1* expression (*p* < 0.01; Figure 2B) and was therefore used in subsequent knockdown experiments. CCK-8 assays showed no significant difference in cell viability among groups at 24 h after transfection (*p* > 0.05). In contrast, at 48 h and 72 h, cell viability was significantly higher in the *YAP1* overexpression group than in the empty-vector group (*p* < 0.05), whereas *YAP1* silencing significantly reduced cell viability relative to the si-NC group (*p* < 0.05; Figure 2C,D). RT-qPCR further showed that *YAP1* overexpression was accompanied by significantly decreased *BAX* expression and significantly increased *PCNA* and *BCL2* expression (*p* < 0.05). Conversely, *YAP1* silencing resulted in significantly increased *BAX* expression and significantly decreased *PCNA* and *BCL2* expression compared with the negative control group (*p* < 0.05; Figure 2E,F).

Overall, these data indicate that altered *YAP1* expression was associated with changes in CCK-8-based Sertoli cell viability and with corresponding mRNA-level changes in viability-associated genes. Because *BAX*, *BCL2*, and *PCNA* were not examined at the protein level, these findings should be interpreted as transcriptional evidence rather than direct evidence of altered proliferation or apoptosis.

### 3.3. Effect of YAP1 Silencing or Overexpression on Hippo Pathway Downstream Genes

**Figure 3:**
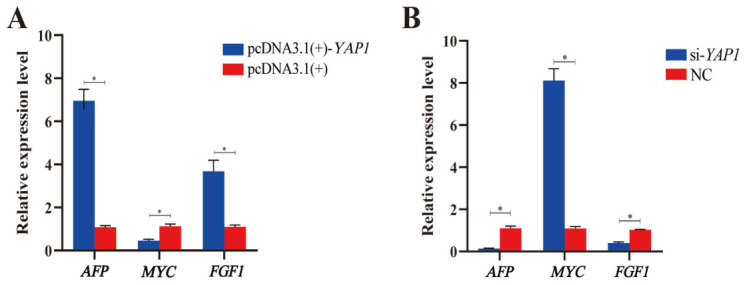
Association of *YAP1* overexpression and silencing with the mRNA expression of selected *YAP1*-related genes in Sertoli cells. ((**A**) RT-qPCR analysis of *AFP*, *MYC* and *FGF1* mRNA expression after transfection with the *YAP1* overexpression plasmid pcDNA3.1(+)-*YAP1* or the empty vector pcDNA3.1(+). (**B**) RT-qPCR analysis of *AFP*, *MYC* and *FGF1* mRNA expression after transfection with si-*YAP1* or the negative control (NC). Data are presented as mean ± SD from three independent biological replicates. Statistical significance in each panel was assessed by Student’s *t*-test between the two indicated groups. differences *AFP*, *MYC* and *FGF1* mRNA expression was measured by RT-qPCR. Data are presented as mean ± SD from three independent biological replicates. Panels (**A**,**B**) were analyzed using an unpaired Student’s *t*-test between the two indicated groups for each gene. Data are presented as mean ± SD. * *p* < 0.05 indicate statistically significant.

RT-qPCR analysis performed 48 h after transfection showed that altered *YAP1* expression was accompanied by changes in several Hippo pathway-related transcripts. In the *YAP1* overexpression group, *AFP* and *FGF1* expression levels were significantly increased compared with those in the empty-vector group (*p* < 0.05), whereas *MYC* expression showed a decreasing trend (Figure 3A). In contrast, *YAP1* silencing significantly reduced *AFP* and *FGF1* expression relative to the control group (*p* < 0.05), while MYC expression was increased (Figure 3B). Overall, these results indicate that *YAP1* modulation was associated with differential transcriptional changes in Hippo pathway-related genes in sheep Sertoli cells, with *AFP* and *FGF1* showing expression patterns more consistent with *YAP1* abundance.

### 3.4. Effect of YAP1 Overexpression or Silencing on Functional Genes in Sheep SCs

**Figure 4:**
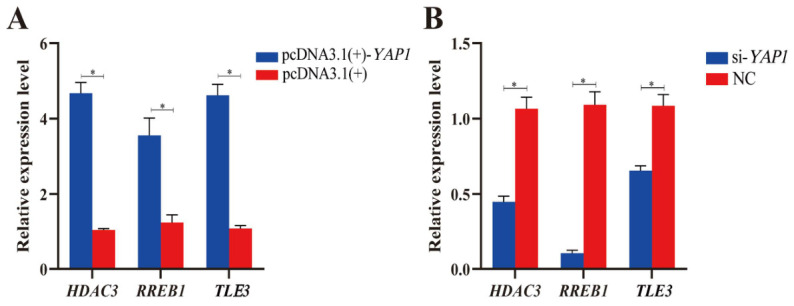
Effects of *YAP1* overexpression and silencing on the expression of Sertoli cell functional genes. (**A**) RT-qPCR analysis of *HDAC3*, *RREB1* and *TLE3* mRNA expression after transfection with the *YAP1* overexpression plasmid pcDNA3.1(+)-*YAP1* or the empty vector pcDNA3.1(+). (**B**) RT-qPCR analysis of *HDAC3*, *RREB1* and *TLE3* mRNA expression after transfection with si-*YAP1* or the negative control (NC). Data are presented as mean ± SD from three independent biological replicates. Statistical significance in each panel was assessed by Student’s *t*-test between the two indicated groups. Data are presented as mean ± SD. * *p* < 0.05 indicate statistically significant, *HDAC3*, *RREB1* and *TLE3* mRNA expression was measured by RT-qPCR. Data are presented as mean ± SD from three independent biological replicates. Panels (**A**,**B**) were analyzed using an unpaired Student’s *t*-test between the two indicated groups for each gene.

RT-qPCR analysis performed 48 h after transfection showed that *YAP1* modulation was associated with changes in the expression of SC functional genes. Compared with the empty-vector group, *HDAC3*, *RREB1* and *TLE3* expression levels were significantly increased in the pcDNA3.1(+)-*YAP1* group (*p* < 0.05; Figure 4A). In contrast, silencing of *YAP1* significantly reduced the expression of these three genes relative to the negative control group (*p* < 0.05; Figure 4B). These results indicate that altered *YAP1* expression was associated with corresponding transcriptional changes in SC functional genes.

### 3.5. miR-132-y Is Associated with YAP1 Expression Through Interaction with the YAP1 3′UTR

**Figure 5:**
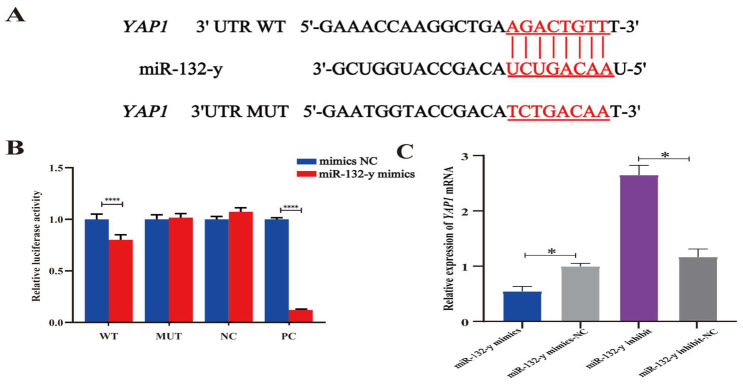
Assessment of the interaction between miR-132-y and the *YAP1* 3′UTR. (**A**) Predicted binding site of miR-132-y within the ovine *YAP1* 3′UTR and the corresponding mutant sequence used for luciferase reporter construction. Red letters indicate the predicted seed-matching region. (**B**) Dual-luciferase reporter assay showing relative luciferase activity in HEK-293T cells co-transfected with miR-132-y mimic or mimic negative control (mimics NC) and reporter constructs containing the wild-type (WT) or mutant (MUT) *YAP1* 3′UTR. NC indicates the negative control reporter, and PC indicates the positive control used in the dual-luciferase assay. (**C**) RT-qPCR analysis of *YAP1* mRNA expression after transfection of sheep Sertoli cells with miR-132-y mimic, mimic negative control, miR-132-y inhibitor, or inhibitor negative control. Data are presented as mean ± SD from three independent biological replicates. In panel B, statistical significance was assessed by one-way ANOVA followed by a post hoc multiple-comparison test. In panel (**C**), statistical significance was assessed by Student’s *t*-test between the two indicated groups. Data are presented as mean ± SD. ns, not significant; * *p* < 0.05; **** *p* < 0.0001 (**B**) was performed using a dual-luciferase reporter assay in HEK-293T cells. Panel (**C**) shows *YAP1* mRNA expression measured by RT-qPCR in sheep Sertoli cells. The miR-132-y mimic was used to increase miR-132-y levels, and mimic NC indicates the corresponding negative control mimic. The miR-132-y inhibitor was used to suppress endogenous miR-132-y activity, and inhibitor NC indicates the corresponding inhibitor negative control. Data are presented as mean ± SD from three independent biological replicates. Panel (**B**) was analyzed using one-way ANOVA followed by Tukey’s post hoc test. Panel (**C**) was analyzed using an unpaired Student’s *t*-test between the two indicated groups.

Previous sequencing analysis identified several candidate miRNAs targeting *YAP1*, among which miR-132-y was selected for further investigation. TargetScan analysis predicted a conserved miR-132-y binding site within the 3′UTR of ovine *YAP1* (Figure 5A). To verify this interaction, luciferase reporter plasmids containing either the wild-type (WT) or mutant (MUT) *YAP1* 3′UTR were constructed. Dual-luciferase reporter assays showed that transfection of miR-132-y mimic significantly reduced luciferase activity in the WT group (*p* < 0.01), whereas no significant change was observed in the MUT group (*p* > 0.05; Figure 5B). In addition, RT-qPCR analysis showed that transfection of the miR-132-y mimic significantly decreased *YAP1* mRNA expression, whereas transfection of the miR-132-y inhibitor significantly increased *YAP1* expression compared with the corresponding control group (*p* < 0.05; Figure 5C). Together, these findings support a direct interaction between miR-132-y and the *YAP1* 3′UTR and indicate that miR-132-y is associated with reduced *YAP1* expression in sheep Sertoli cells.

### 3.6. Association of miR-132-y Modulation with Sertoli Cell Viability and Viability-Related Transcript Expression

**Figure 6:**
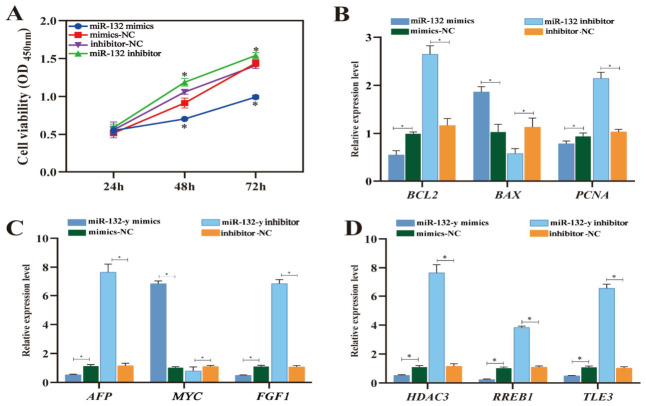
Effects of miR-132-y on Sertoli cell viability and gene expression. (**A**) CCK-8 assay showing changes in Sertoli cell viability at 24, 48, and 72 h after transfection with miR-132-y mimic, mimic negative control (mimics-NC), miR-132-y inhibitor, or inhibitor negative control (inhibitor-NC). (**B**) RT-qPCR analysis of *BCL2*, *BAX* and *PCNA* mRNA expression after transfection with miR-132-y mimic, mimics-NC, miR-132-y inhibitor, or inhibitor-NC. (**C**) RT-qPCR analysis of selected YAP1-related transcripts, including *AFP*, *MYC* and *FGF1*, after transfection with miR-132-y mimic, mimic-NC, miR-132-y inhibitor, or inhibitor-NC. (**D**) RT-qPCR analysis of Sertoli cell functional genes, including *HDAC3*, *RREB1* and *TLE3*, after transfection with miR-132-y mimic, mimic-NC, miR-132-y inhibitor, or inhibitor-NC. Panel (**A**) shows Sertoli cell viability measured by CCK-8 assay at 24, 48 and 72 h after transfection. Panels (**B**–**D**) show mRNA expression measured by RT-qPCR. The miR-132-y mimic was used to increase miR-132-y levels, and mimic NC indicates the corresponding negative control mimic. The miR-132-y inhibitor was used to suppress endogenous miR-132-y activity, and inhibitor NC indicates the corresponding inhibitor negative control. Data are presented as mean ± SD from three independent biological replicates. Panel (**A**) was analyzed by comparing the indicated groups at each time point. Panels (**B**–**D**) were analyzed using one-way ANOVA followed by Tukey’s post hoc test. Data are presented as mean ± SD. * *p* < 0.05 indicate statistically significant.

To evaluate the effects of miR-132-y on sheep Sertoli cells, cell viability was first assessed using the CCK-8 assay. No significant differences were observed between the miR-132-y overexpression or inhibition groups and their corresponding control groups at 24 h post-transfection. However, at 48 h and 72 h, cell viability was significantly lower in the miR-132-y mimic group than in the control group, whereas transfection of the miR-132-y inhibitor significantly increased cell viability relative to the corresponding control group (Figure 6A). RT-qPCR was subsequently performed to examine transcriptional changes in proliferation- and apoptosis-related genes. Overexpression of miR-132-y significantly increased *BAX* expression and significantly decreased *PCNA* and *BCL2* expression (*p* < 0.05). In contrast, inhibition of miR-132-y produced the opposite expression pattern (Figure 6B). Taken together, these results indicate that miR-132-y modulation was associated with changes in CCK-8-based Sertoli cell viability and with altered mRNA expression of viability-associated genes. These data do not directly demonstrate changes in proliferation or apoptosis, which require further validation using protein-level and cell-based functional assays. Since the corresponding protein levels of *BAX*, *BCL2* and *PCNA* were not measured, the effects of miR-132-y on proliferation- or apoptosis-related proteins remain to be determined.

### 3.7. Impact of miR-132-y on Hippo Pathway Downstream Targets and Functional Genes in SCs

RT-qPCR analysis showed that miR-132-y modulation was associated with changes in the expression of Hippo/YAP signaling-related genes. Compared with the corresponding control group, *AFP*, *MYC* and *FGF1* expression levels were significantly decreased in the miR-132-y mimic group (*p* < 0.05), whereas the miR-132-y inhibitor group showed the opposite pattern (*p* < 0.05; Figure 6C). A similar trend was observed for SC functional genes. Relative to the corresponding control group, *HDAC3*, *RREB1* and *TLE3* expression levels were reduced in the miR-132-y mimic group and significantly increased in the miR-132-y inhibitor group (Figure 6D). Because *YAP1* protein abundance, YAP phosphorylation status, *YAP* subcellular localization, and *YAP*/*TAZ*-TEAD transcriptional reporter activity were not assessed, these data should be interpreted as transcript-level evidence of selected *YAP1*-related responses rather than direct evidence of altered Hippo/*YAP* pathway activity.

### 3.8. Rescue Experiment

**Figure 7:**
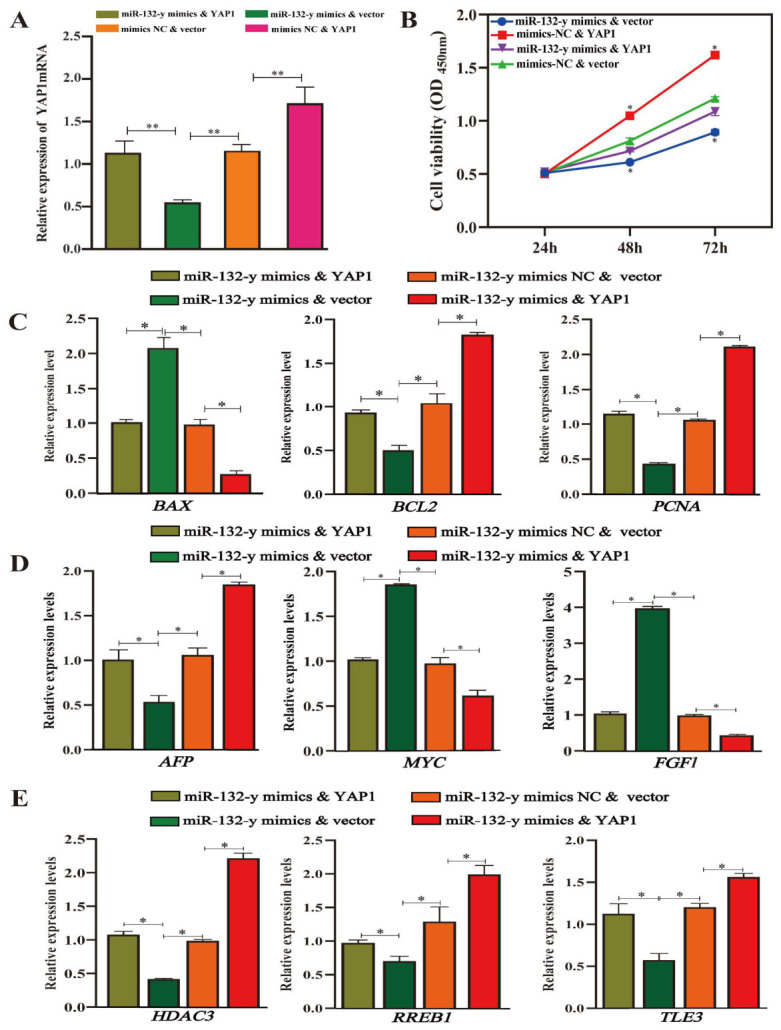
Rescue Experiment. (**A**) *YAP1* mRNA expression levels following co-transfection. (**B**) Cell viability assessed by CCK-8 assay. (**C**) Expression of proliferation- and apoptosis-related genes (*BAX*, *BCL2*, and *PCNA*). (**D**) RT-qPCR analysis of selected *YAP1*-related transcripts, including *AFP*, *MYC* and *FGF1*, in the rescue experiment. (**E**) Expression of functional genes (*HDAC3*, *RREB1*, and *TLE3*). Panel (**A**) shows *YAP1* mRNA expression measured by RT-qPCR after co-transfection. Panel (**B**) shows Sertoli cell viability measured by CCK-8 assay. Panels (**C**–**E**) show mRNA expression measured by RT-qPCR. The rescue experiment included four groups: miR-132-y mimic + pcDNA3.1(+)-*YAP1*, miR-132-y mimic + empty vector, mimic NC + pcDNA3.1(+)-*YAP1*, and mimic NC + empty vector. The miR-132-y mimic was used to increase miR-132-y levels; mimic NC indicates the corresponding negative control mimic; pcDNA3.1(+)-*YAP1* indicates the *YAP1* overexpression plasmid; and empty vector indicates pcDNA3.1(+). Data are presented as mean ± SD from three independent biological replicates. Panels (**A**–**E**) were analyzed using one-way ANOVA followed by Tukey’s post hoc test. * *p* < 0.05 was considered statistically significant; ** *p* < 0.01.

To further assess whether YAP1 mediates the effects of miR-132-y in sheep Sertoli cells, a rescue experiment was performed using four transfection groups: miR-132-y mimic + pcDNA3.1(+)-*YAP1*, miR-132-y mimic + empty vector, mimic negative control + pcDNA3.1(+)-*YAP1* and mimic negative control + empty vector. RT-qPCR analysis showed that *YAP1* mRNA expression in the co-transfection group was significantly higher than that in the miR-132-y mimic + empty vector group (*p* < 0.05), whereas no significant difference was observed relative to the negative control group (*p* > 0.05; Figure 7A).

Compared with the miR-132-y mimic + empty vector group, co-transfection with the *YAP1* overexpression plasmid significantly increased cell viability and was accompanied by expression changes in proliferation- and apoptosis-related genes opposite to those induced by miR-132-y overexpression alone (Figure 7B,C). In addition, the altered expression of Hippo pathway-related genes and SC functional genes induced by miR-132-y overexpression was partially attenuated by ectopic YAP1 expression (Figure 7D,E).

## 4. Discussion

Although primary Sertoli cell culture systems have been well established in rodents, species-specific differences remain an important consideration when isolating and culturing testicular somatic cells from livestock and poultry species [19,20,21,22,23,24,25]. Previous methodological studies have shown that mechanical dissociation combined with enzymatic digestion is an effective strategy for Sertoli cell isolation, and marker-based characterization is necessary to evaluate cell identity and enrichment [26,27]. In the present study, primary Sertoli cells were enriched from 4-month-old Southdown × Hu F1 sheep testes using enzymatic dissociation, hypotonic treatment, and differential adhesion, based on previously described principles for Sertoli cell preparation and identification [28,29,30,31,32,33,34]. The isolated cells showed typical elongated or bipolar morphology and were characterized by Oil Red O staining, weak alkaline phosphatase staining, and positive immunofluorescence signals for the Sertoli cell markers *GATA4* and *SOX9*, with an estimated purity exceeding 85%. These results indicate that the culture system was enriched for Sertoli cells and suitable for subsequent in vitro assays. Nevertheless, because a comprehensive negative-marker panel for germ cells, Leydig cells, and peritubular myoid cells was not performed, minor contamination by other testicular cell populations cannot be completely excluded. Therefore, the observed responses should be interpreted in the context of an enriched primary Sertoli cell culture system rather than as definitive Sertoli cell-autonomous effects.

A limitation of this study is that cell proliferation and apoptosis were inferred from gene expression and viability assays rather than directly quantified at the cellular level. The dynamic balance between cell proliferation and apoptosis is precisely regulated by a multilevel regulatory network, involving the synergy between cyclins (Cyclins/CDKs) and apoptosis-related molecules (Bcl2 family/Caspases). Proliferating cell nuclear antigen (*PCNA*) plays an important role in DNA replication and, together with cyclin-dependent kinases, contributes to G1/S-phase progression and cell proliferation [35]. As a representative pro-apoptotic member of the *BCL2* family, *BAX* participates in the mitochondrial apoptosis pathway. In response to apoptotic stimuli, *BAX* translocates to mitochondria and contributes to mitochondrial outer membrane permeabilization. This process promotes the release of pro-apoptotic factors, such as cytochrome c, and activates the caspase cascade [36]. Overexpression of *BAX* can induce cell apoptosis. Bax-deficient male mice exhibit infertility accompanied by abnormal seminiferous tubule architecture and the accumulation of atypical premeiotic germ cells. This abnormal condition hinders the production of mature sperm and ultimately leads to atrophy of the testicles in adult mice [37]. The *BAX* gene plays a role in the process of spermatogenesis and in maintaining the number of cells in the testicle environment. The *BAX* defect leads to a large accumulation of germ cells before meiosis in mature animals, and almost complete absence of sperm cells and mature sperm [38]. After Xi et al. performed local testicular heating of pig testes in vitro, mRNA and protein levels of *BAX* and *Bcl2* increased, resulting in an increase in apoptotic germ cells [39]. Zhao et al. administered fluoride treatment to rats and assessed the expression of the proliferation markers *PCNA* and Ki-67 in testicular and epididymal tissues using immunohistochemistry. F treatment significantly increased the apoptosis of spermatogenetic cells in the testicles. It was found that *PCNA* and Ki-67 were also positively expressed in the testicles, resulting in spermatogenesis disorders [40,41]. When *YAP1* is overexpressed in SCs, the expression level of *BAX* is downregulated, while the expression levels of *PCNA* and *Bcl2* are upregulated; on the contrary, after interfering with *YAP1* expression, the expression level of *BAX* is increased, and the expression of *PCNA* is suppressed. The relatively modest changes in cell viability observed in this study may reflect the tightly controlled nature of Sertoli cell proliferation and survival, where subtle regulatory effects can still be biologically meaningful. These results suggest that *YAP1* modulation is associated with changes in CCK-8-based cell viability and in the mRNA expression of selected viability-associated genes. However, because direct proliferation/apoptosis assays and protein-level validation were not performed, these findings should not be interpreted as definitive evidence that *YAP1* directly promotes proliferation or inhibits apoptosis.

The Hippo signaling pathway, as a signal pathway that regulates cell proliferation, differentiation and survival, mainly plays a role in cancer and tumor diseases. Alpha-fetoprotein (*AFP*), a diagnostic marker for non-seminomatous germ cell tumors and testicular cancer prior to treatment, was positive in more than 50% of patients in the study by Takami et al. [42]. After overexpressing *YAP1* in this study, the *AFP* expression level was significantly upregulated, and it was speculated that the increase in its expression level was related to the activation of *YAP1*. After silencing, the *AFP* expression level was significantly reduced, which further evidenced the speculation of this study. The Hippo signaling pathway also plays an important role in maintaining dynamic tissue balance and organ regeneration. After liver damage, the expression of *FGF1* increases by activating the Hippo signaling pathway, promoting the proliferation of hepatocytes and liver regeneration [43].

Sertoli cells are essential for germ cell development by supporting maturation and establishing the blood–testis barrier (BTB), which provides an immune-protective microenvironment for meiotic and postmeiotic germ cells [44]. Among the Sertoli cell function-associated genes examined in this study, *HDAC3* has been implicated in germ cell development and Sertoli cell maturation-related epigenetic regulation [45,46]. *RREB1*, a conserved zinc finger transcription factor, is enriched in Sertoli cells and has been associated with Sertoli cell development and FSHR promoter activity, suggesting a potential role in spermatogenesis-related signaling [47,48,49,50]. *TLE3*, a transcriptional corepressor, also shows stage- and cell type-associated expression during male germ cell development, and altered *TLE3* expression has been linked to abnormal Sertoli cell responses [51,52,53]. In the present study, *YAP1* overexpression was accompanied by increased *HDAC3*, *RREB1* and *TLE3* mRNA expression, whereas *YAP1* silencing showed the opposite pattern. These findings suggest that *YAP1* modulation is associated with transcriptional changes in selected Sertoli cell function-associated genes. However, because these genes were measured only at the mRNA level, and *YAP1* protein abundance, phosphorylation status, subcellular localization, and transcriptional activity were not directly assessed, these results should be interpreted as transcript-level associations rather than direct evidence that *YAP1* regulates BTB function or Sertoli cell maturation.

Previous studies have demonstrated that follicle-stimulating hormone (FSH) can regulate YAP activity in Sertoli cells, highlighting the interaction between hormonal signaling and the Hippo pathway. For example, FSH has been shown to influence YAP phosphorylation status and its downstream transcriptional activity in cultured Sertoli cells [54,55]. Although FSH signaling was not directly investigated in the present study, our findings that miR-132-y regulates *YAP1* expression suggest a potential link between post-transcriptional regulation and hormone-mediated signaling pathways. These observations imply that *YAP1* regulation in Sertoli cells may involve complex interactions between miRNA-mediated mechanisms and endocrine signaling.

MicroRNAs regulate gene expression post-transcriptionally by binding to complementary sequences within the 3′UTR of target mRNAs, leading to mRNA degradation and translational repression [56]. In the present study, the dual-luciferase reporter assay and RT-qPCR results support a direct regulatory relationship between miR-132-y and the *YAP1* 3′UTR in sheep Sertoli cells. Specifically, miR-132-y overexpression decreased *YAP1* mRNA abundance, whereas miR-132-y inhibition increased *YAP1* expression, indicating that *YAP1* is a transcript-level target of miR-132-y in this in vitro model. However, the effects of miR-132-y are unlikely to be mediated exclusively through *YAP1*. Because a single miRNA can regulate multiple downstream mRNAs simultaneously, the changes in CCK-8-based cell viability and viability-associated transcript expression observed after miR-132-y modulation may reflect the combined contribution of *YAP1*-dependent and *YAP1*-independent regulatory events [57]. Therefore, the present findings should be interpreted as evidence that miR-132-y is associated with a post-transcriptional regulatory network involving *YAP1*, rather than as proof of a single linear miR-132-y–*YAP1* pathway. Future studies using transcriptome-wide target identification, protein-level validation, and direct functional assays will be required to define the broader target spectrum of miR-132-y and its role in Sertoli cell biology.

Several limitations of this study should be acknowledged. First, all functional analyses were performed in an in vitro primary Sertoli cell culture system, and the physiological relevance of the miR-132-y–*YAP1* relationship remains to be validated in vivo. Second, the present study mainly relied on CCK-8 assays and RT-qPCR analysis of selected transcripts. CCK-8 reflects cellular metabolic activity and overall viability, but it does not directly distinguish proliferation from apoptosis. Similarly, changes in *BAX*, *BCL2*, *PCNA*, *AFP*, *MYC*, *FGF1*, *HDAC3*, *RREB1* and *TLE3* mRNA abundance do not necessarily indicate corresponding changes at the protein or functional level. Third, *YAP1* protein abundance, YAP phosphorylation status, YAP subcellular localization, and YAP/TAZ-TEAD transcriptional activity were not directly examined. Fourth, although the isolated cell population was enriched for Sertoli cells based on morphology, Oil Red O staining, weak ALP staining, and *GATA4*/*SOX9* immunofluorescence, a comprehensive negative-marker panel for germ cells, Leydig cells, and peritubular myoid cells was not performed. Therefore, minor contamination by other testicular cell types cannot be completely excluded. These limitations indicate that the current findings should be interpreted as cell-based evidence for a miR-132-y–*YAP1* regulatory relationship and associated transcriptional responses, rather than definitive proof of an in vivo Sertoli cell-autonomous mechanism.

MiRNAs exhibit dynamic expression patterns during male reproductive development and have been implicated in the regulation of spermatogenesis and Sertoli cell function. Taking miR-361-3p as an example, this molecule specifically regulates the expression level of the FSHB gene, thereby affecting the biosynthesis process of gonadotropin, and ultimately participates in the biological mechanism of regulating spermatogenesis [58]. Another study found that miR-762 can effectively stimulate the proliferation activity of immature Sertoli cells by acting on the *RNF4* gene regulatory network in the pig model, which provides a new perspective for the study of the mechanism of germ cell development [59]. The Hippo signaling pathway is a mechanism that regulates the size of mammalian and human organs. Organ size is regulated by mediating cell growth, division, and death. In the case of low cell density, inhibition of the Hippo pathway leads to activation of YAP, and the activated YAP moves toward the nucleus. This series of events leads to the activation of subsequent genes responsible for elevated cell growth, thereby inhibiting the biogenesis of miRNAs [60]. In addition, miRNAs can target components of the Hippo pathway. miR-132 has been reported to show differential expression between embryonic and postnatal sperm, and sperm concentration has been positively associated with ANO1 expression in sperm [61]. Functional studies have shown that bta-miR-146b overexpression inhibits the proliferation of bovine male germline stem cells and may participate in apoptosis-related regulation. In contrast, regulating bta-miR-146b inhibitors can promote cattle reproduction and proliferation, resulting in stagnation of yak sperm [62]. In cynomolgus monkeys, altered miR-34b-5p and miR-34c-5p expression has been associated with changes in germ cell number, including reductions in spermatocytes and spermatids [63]. Another study reported a negative association between miR-29a and TRPV4 expression during testicular ischemia–reperfusion injury, with reduced miR-29a expression observed in both animal tissues and GC-1 germ cells. MiR-29a inhibition upregulates *TRPV4* and aggravates germ cell apoptosis, while overexpression significantly reduces *TRPV4* levels, effectively alleviating IRI-induced apoptosis in vivo and in vivo models [64].

**Figure 8:**
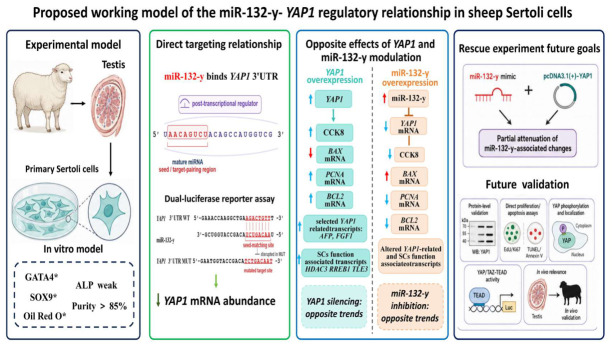
Proposed working model of the miR-132-y–*YAP1* regulatory relationship in sheep Sertoli cells. Primary Sertoli cells isolated from Southdown × Hu F1 sheep were used as an in vitro model. miR-132-y was predicted and experimentally supported to interact with the *YAP1* 3′UTR by target prediction and dual-luciferase reporter assays. *YAP1* overexpression and miR-132-y overexpression showed opposite associations with CCK-8-based cell viability and with the mRNA expression of selected viability-associated, *YAP1*-related, and Sertoli cell function-associated genes. Rescue experiments further showed that ectopic *YAP1* expression partially attenuated miR-132-y-associated changes, * refers to cell signature antibody markers.

Although the present study provides in vitro, cell-based evidence supporting a regulatory relationship between miR-132-y and *YAP1* in sheep Sertoli cells, several additional experiments should be performed to further strengthen and extend the current findings. First, protein-level validation is required, including the detection of *YAP1*, phosphorylated *YAP*, *BAX*, *BCL2*, *PCNA*/*Ki67*, cleaved caspase-3, and cleaved *PARP*, to determine whether the observed mRNA-level changes are translated into corresponding protein-level alterations. Second, direct functional assays, such as EdU or Ki67 staining, TUNEL staining, and Annexin V/PI flow cytometry, are necessary to distinguish effects on proliferation and apoptosis from general changes in CCK-8-based cell viability. Third, YAP subcellular localization, YAP phosphorylation status, YAP/TAZ-TEAD reporter activity, and additional canonical YAP/TAZ target genes should be examined to determine whether miR-132-y functionally modulates Hippo-YAP signaling activity. Fourth, the upstream mechanisms regulating miR-132-y expression in Sertoli cells, including endocrine, developmental, and stress-related signals, should be investigated. Finally, in vivo studies in developing testes and further analyses linking this regulatory axis to male reproductive traits will be important for defining its physiological relevance to testicular development, spermatogenesis, mammalian reproductive biology, and livestock breeding (Figure 8).

## 5. Conclusions

In summary, this study provides in vitro, cell-based evidence that *YAP1* is a transcript-level target of miR-132-y in sheep Sertoli cells. The predicted miR-132-y binding site within the *YAP1* 3′UTR was supported by a dual-luciferase reporter assay, and miR-132-y modulation was associated with reciprocal changes in *YAP1* mRNA abundance. Gain- and loss-of-function analyses showed opposite associations between *YAP1* or miR-132-y modulation and CCK-8-based Sertoli cell viability, as well as the mRNA expression of selected viability-associated, *YAP1*-related, and Sertoli cell function-associated genes. Rescue experiments further showed that ectopic *YAP1* expression partially attenuated miR-132-y-associated changes, suggesting that *YAP1* may contribute, at least in part, to miR-132-y-associated transcriptional responses in this in vitro model.

Collectively, these findings extend the miRNA–*YAP1* regulatory framework to ovine primary Sertoli cells and provide a basis for further investigation of post-transcriptional regulation in testicular somatic cells. To strengthen the results presented in this study and further define the biological significance of the miR-132-y–*YAP1* axis, additional experiments should be performed in future work, including protein-level validation, direct proliferation and apoptosis assays, YAP phosphorylation and subcellular localization analyses, YAP/TAZ-TEAD activity assays, upstream regulatory assessment of miR-132-y, and in vivo verification in developing testes. These additional studies will be important for determining the physiological relevance of this regulatory axis to Sertoli cell function, testicular development, spermatogenesis, and male reproductive performance.

## Data Availability

The original contributions presented in this study are included in the article/supplementary material. Further inquiries can be directed to the corresponding authors.

## Supplementary Materials

The following supporting information can be downloaded at: https://www.mdpi.com/article/10.3390/biom16070995/s1, Figure S1: Complete nucleic acid sequence information of YAP1 3′-UTR wild-type and mutant recombinant plasmids; Table S1: Information of si-*YAP1* sequence; Table S2: Information of miRNA sequence; Table S3: Information of primer sequence.
