# Supplementary material for: miR-132-y Targets YAP1 and Modulates Sertoli Cell Viability-Associated Transcriptional Responses in Southdown × Hu F1 Sheep
Source: Biomolecules. 2026 Jul 7;16(7):995. doi: 10.3390/biom16070995 (PMC13406466; doi:10.3390/biom16070995)
Supplement: Supplementary file 1 [file biomolecules-16-00995-s001.zip › biomolecules-4360889-supplementary.pdf]

YAP1-miR-132-y -WT：5’-
CCAGTGGGAAAACATGACTTACTGGTCTAACAAGCCAAAAATGTTGTATC
TGATGTTTAGTACTTAGACTGATTTGAAGAGCTAGCTGAAACCAAGGCTGA
AGACTGTTTTACTTTCAGTGGGTTTTTTTTCCTCCTAGTGCTATCATTAGTCA
CATAGTGACCTTGATTTTATTTTAGGAG**CTTCTAAGGCTTGAGATA-3’**
YAP1-miR-132-y-MUT：5’-
CCAGTGGGAAAACATGACTTACTGGTCTAACAAGCCAAAAATGTTGTATC
TGATGTTTAGTACTTAGACTGATTTGAAGAGCTAGCTGAATGGTACCGACAT
CTGACAATTACTTTCAGTGGGTTTTTTTTCCTCCTAGTGCTATCATTAGTCAC
ATAGTGACCTTGATTTTATTTTAGGAG**CTTCTAAGGCTTGAGATA-3’**

Figure S1. Complete nucleic acid sequence information of *YAP1* 3'-UTR wild-type and mutant recombinant plasmids.

Table S1. Information of si-*YAP1* sequence

Name	Sequence	
	Sense (5'-3')	Antisense (5'-3')
si-*YAP1*-1	GGUCAGAGGUACUUCUUAATT	UUAAGAAGUACCUCUGACCTT
si-*YAP1*-2	GGUGACACUAUCAACCAAATT	UUUGGUUGAUAGUGUCACCTT
si-*YAP1*-3	CACCAAGCUAGAUAAAGAATT	UUCUUUAUCUAGCUUGGUGTT
si-*YAP1*-NC	CACUAGAUAAACAATTGCAAG	CACACAATTGCAAGUAGAUAA

Table S2. Information of miRNA sequence

Name	Sequence	
	Sense (5'-3')	Antisense (5'-3')
miR-132-y mimics	UAACAGUCUACAGCCAUGGUCG	ACCAUGGCUGUAGACUGUUAUU
mimics NC	UUCUCCGAACGUGUCACGUTT	ACGUGACACGUUCGGAGAATT
miR-132-y inhibitor	CGACCAUGGCUGUAGACUGUUA	
inhibitor NC	CAGUUUUAGUACAAAGUGUCU	

Table S3. Information of primer sequence

Primer name	Forward primer (5'-3')	Reverse primer (5'-3')
YAP1	CAGTTCCAACCAGCAGCAAC	AGCTAATTCCTGCCGAAGCA
BCL2	ATGTGTGTGGAGAGCGTCAA	CCATGTTTTGATTTCCCAGCCTC
BAX	CAGAGGCGGGGTTTCATCC	CTTCAGACACTCGCTCAGCTT
PCNA	TGCAGATGTACCCCTTGTTGT	CATCCTCGATCTTGGGAGCC
HDAC3	ATTCGAGGACACGGGGAATG	ACCACCACCTAGCACCAGTA
RREB1	CTCCAGAGGCGGTGGCTTT	CGTATCTCTCGTTGACACTGTCTTA
TLE3	GCATGGACCCGATAGCCTC	TGGTGGCTCATCATGGCAAA
AFP	CGTGCTGCTTTGGGAGTCTA	AATGCTAGCTCTTGGGATGC
MYC	GTCACTCAGAGCGGGCTAAA	TGCACCGAATCGTAGTCGAG
FGF1	AGGCTGTCTCAGGATGACCT	GCCTGCTCCCCTCAGTTAAG
β-actin	CTTCCAGCCTTCCTTCCTGG	GCCAGGGCAGTGATCTCTTT
